# Combined Influence of Hall Current and Soret Effect on Chemically Reacting Magnetomicropolar Fluid Flow from Radiative Rotating Vertical Surface with Variable Suction in Slip-Flow Regime

**DOI:** 10.1155/2014/102413

**Published:** 2014-10-30

**Authors:** Preeti Jain

**Affiliations:** Department of Mathematics, University of Rajasthan, Jaipur 302004, India

## Abstract

An analysis study is presented to study the effects of Hall current and Soret effect on unsteady hydromagnetic natural convection of a micropolar fluid in a rotating frame of reference with slip-flow regime. A uniform magnetic field acts perpendicularly to the porous surface which absorbs the micropolar fluid with variable suction velocity. The effects of heat absorption, chemical reaction, and thermal radiation are discussed and for this Rosseland approximation is used to describe the radiative heat flux in energy equation. The entire system rotates with uniform angular velocity Ω about an axis normal to the plate. The nonlinear coupled partial differential equations are solved by perturbation techniques. In order to get physical insight, the numerical results of translational velocity, microrotation, fluid temperature, and species concentration for different physical parameters entering into the analysis are discussed and explained graphically. Also, the results of the skin-friction coefficient, the couple stress coefficient, Nusselt number, and Sherwood number are discussed with the help of figures for various values of flow pertinent flow parameters.

## 1. Introduction

Micropolar fluids are subset of the micromorphic fluid. Micropolar fluids are those fluids consisting of randomly oriented particles suspended in a viscous medium, which can undergo a rotation that can affect the hydrodynamics of the flow, making it a distinctly non-Newtonian fluid. They constitute an important branch of non-Newtonian fluid dynamics where microrotation effects as well as microinertia are exhibited. Modelling and analysis of the dynamics of micropolar fluids have been the field of very active research due to their application in a number of processes that occur in chemical, pharmaceutical, and food industry. Such applications include the extrusion of polymer fluids, solidification of liquid crystals, cooling of a metallic plate in a bath, animal bloods, exotic lubricants, and colloidal and suspension solutions, for example, for which the classical Navier-Stokes theory is inadequate. The essence of the theory of micropolar fluids lies in the extension of the constitutive equations for Newtonian fluids so that more complex fluids can be described by this theory. In this theory, rigid particles contained in a small fluid volume element are limited to rotation about the centre of the volume elements described by microrotation vector. It is well known that heterogeneous mixtures, such as Ferro liquids, colloidal fluids, most slurries, and suspensions, are some liquids with polymer activities which behave differently from Newtonian fluids. The main difference is that these types of fluids have a microstructure and exhibit microrotational effects and can support surface and body couples which are not present in the theory of Newtonian fluids. In order to study such types of fluids Eringen [1] developed the theory of microfluids which include the effect of local rotary inertia, the couple stress, and inertial spin. This theory is expected to be successful in analyzing the behavior of non-Newtonian fluids. Eringen [2] also developed the theory of micropolar fluids for the case where only microrotational effects and microrotational inertia exist. He [3] extended the theory of thermomicropolar fluids and derived the constitutive law for fluids with microstructure. An excellent review of micropolar fluids and their applications was given by Ariman et al. [4]. In view of Lukaszewicz [5], micropolar fluids represent those fluids which consist of randomly oriented particles suspended in a viscous medium.

Several authors have studied the characteristic of the boundary layer flow of micropolar fluid under different boundary conditions. Takhar and Soundalgekar [6, 7] studied the flow and heat transfer of micropolar fluid past a porous plate. Further, they [8, 9] discussed these problems past a continuously moving porous plate. Often experimental and analytical investigations of free convection flows are carried out by the researchers, since in many situations in technology and nature, one continually encounters masses of fluid arising freely in an extensive medium due to the buoyancy effects. Gorla et al. [10, 11] investigated the natural convection from a heated vertical plate in micropolar fluid. The problem of flow and heat transfer for a micropolar fluid past a porous plate embedded in a porous medium has been of great use in engineering studies such as oil exploration and thermal insulation. Raptis and Takhar [12] and Kim [13] have considered the micropolar fluid through a porous medium.

All the above mentioned studies are limited only to applications where radiative heat transfer is negligible. The role of thermal radiation in the flow heat transfer process is of great relevance in the design of many advanced energy conversion systems operating at higher temperatures. Thermal radiation within these systems is usually the result of emission by hot walls and the working fluid. Nuclear power plants, gas turbines, and the various propulsion devices for aircraft, missiles, satellites, and space vehicles are examples of such engineering areas. Perdikis and Raptis [14] illustrated the heat transfer of a micropolar fluid in the presence of radiation. Raptis [15] studied the effect of radiation on the flow of a micropolar fluid past a continuously moving plate. Recently, Elbashbeshy and Bazid [16] and Kim and Fedorov [17] have reported on the radiation effects on the mixed convection flow of micropolar fluid. Makinde [18] examined the transient free convection interaction with thermal radiation of an absorbing emitting fluid along moving vertical permeable plate. Rahman and Sattar [19] studied transient convective flow of micropolar fluid past a continuous moving porous plate in the presence of radiation. Moreover, when the radiative heat transfer takes place, the fluid involved can be electrically conducting in the sense that it is ionized owing to high operating temperature. Accordingly, it is of interest to examine the effect of the magnetic field on the flow. Thermal radiation effects on hydromagnetic natural convection flow with heat and mass transfer play an important role in manufacturing processes taking place in industries for the design of fins, glass production, steel rolling, casting and levitation, furnace design, and so forth. The process of fusing of metals in an electrical furnace by applying a magnetic field and the process of cooling of the first wall inside a nuclear reactor containment vessel where the hot plasma is isolated from the wall by applying a magnetic field are examples of such fields where thermal radiation and magnetohydrodynamics (MHD) are correlative. This fact was taken into consideration by Abd-El Aziz [20] in his study on micropolar fluids. Raptis and Massalas [21] studied magnetohydrodynamic flow past a plate by the presence of radiation.

The rotating flow of an electrically conducting fluid in presence of magnetic field has got its importance in Geophysical problems. Investigation of hydromagnetic natural convection flow in a rotating medium is of considerable importance due to its application in various areas of geophysics, astrophysics, and fluid engineering, namely, maintenance and secular variations in Earth's magnetic field due to motion of Earth's liquid core, internal rotation rate of the sun, structure of the magnetic stars, solar and planetary dynamo problems, turbo machines, rotating MHD generators, rotating drum separators for liquid metal MHD applications, and so forth. It may be noted that Coriolis and magnetic forces are comparable in magnitude and Coriolis force induces secondary flow in the flow-field. Changes that take place in the rotation suggest the possible importance of hydromagnetic spin-up. Taking into consideration the importance of such study, unsteady hydromagnetic natural convection flow past a moving plate in a rotating medium is studied by a number of researchers. Mention maybe made of research studies of Singh [22], Raptist and Singh [23], Tokis [24], Nanousis [25], and Singh et al. [26]. This problem of spin-up in magnetohydrodynamic rotating fluids has been discussed under varied conditions by Takhar et al. [27].

The study of heat and mass transfer due to chemical reaction is also very importance because of its occurrence in most of the branches of science and technology. The processes involving mass transfer effects are important in chemical processing equipment which is designed to draw high value products from cheaper raw materials with the involvement of chemical reaction. Ibrahim and Makinde [28] investigated radiation effect on chemically reactive MHD boundary layer flow of heat and mass transfer past a porous vertical flat plate. Babu and Satya Narayan [29] examined chemical reaction and thermal radiation effects on MHD convective flow in a porous medium in the presence of suction. Das [30] and Sivaiah [31] investigated studied the effect of chemical reaction and thermal radiation on heat and mass transfer flow of MHD micropolar aid in a rotating frame of reference. Convection problems associated with heat sources within fluid-saturated porous media are of great practical significance in geophysics and energy-related problems, such as recovery of petroleum resources, cooling of underground electric cables, storage of nuclear waste materials groundwater pollution, fiber and granular insulations, chemical catalytic reactors, and environmental impact of buried heat generating waste. Bakr et al. [32, 33] presented an analysis on MHD free convection and mass transfer adjacent to moving vertical plate for micropolar fluid in a rotating frame of reference in presence of heat generation/absorption and a chemical reaction using perturbation technique. Babu and Narayana [34] analyzed unsteady free convection with heat and mass transfer flow for a micropolar fluid through a porous medium with a variable permeability bounded by a semi-infinite vertical plate in the presence of heat generation, thermal radiation and first-order chemical reaction.

In all this study, the effect of Hall current is not considered. The current development of magnetohydrodynamics application is toward a strong magnetic field (so that the influence of electromagnetic force is noticeable) and toward a low density of the gas (such as in space flight and in nuclear fusion research). Under this condition, the Hall current becomes important. The rotating flow of an electrically conducting fluid in the presence of a magnetic field is encountered in cosmic fluid dynamics, medicine and biology. Application in biomedical engineering includes cardiac MRI, and ECG. MHD was pioneered Cowling [35] and he emphasized that when the strength of the applied magnetic field is sufficiently large, Ohm's law needs to be modified to include Hall current. The Hall effect is merely due to the sideways magnetic force on the drafting free charges. The electric field has to have a component transverse to the direction of the current density to balance this force. In many works of plasma physics, much attention is not paid to the effect caused due to Hall current. However, the Hall Effect cannot be completely ignored if the strength of the magnetic field is high and the number of density of electrons is small as it is responsible for the change of the flow pattern of an ionized gas. Hall effect results in a development of an additional potential difference between opposite surfaces of a conductor for which a current is induced perpendicular to both the electric and magnetic field. This current is termed as Hall current. Deka [36], Takhar et al. [37], Saha et al. [38], and Ahmed et al. [39] have presented some model studies on the effect of Hall current on MHD convection flow because of its possible application in the problem of MHD generators and Hall current. Preeti and Chaudhary [40] analyzed an unsteady hydromagnetic flow of a viscoelastic fluid from a radiative vertical porous plate, taking the effects of Hall current and mass transfer into account. Kinyanjui et al. [41] studied the heat and mass transfer in unsteady free convection flow with radiation absorption past an impulsively started infinite vertical porous plate subjected to strong magnetic field including the Hall effect. Takhar et al. [42] investigated the simultaneous effects of Hall current and free stream velocity on the magneto hydrodynamic flow over a moving plate in a rotating fluid. Recently, Seth et al. [43] investigated the problem of an unsteady MHD free convective flow past an impulsively started vertical plate with ramped temperature immersed in a porous medium with rotation and heat absorption taken into account the Hall Effect.

When heat and mass transfer occur simultaneously in a moving fluid, the relations between the fluxes and the driven potential are important. It has been found that an energy flux can be generated not only by temperature gradients but by composition gradient as well. The energy caused by a composition gradient is called the Dufour or the diffusion-thermo effect, also the mass fluxes can also be caused by the temperature gradient and this is called the Soret or thermal diffusion effect; that is, if two regions in a mixture are maintained at different temperatures so that there is a flux of heat, it has been found that a concentration gradient is set up and in a binary mixture, one kind of a molecule tends to travel toward the hot region and the other kind toward the cold region. This is called the “Soret effect.” The Dufour effect is neglected in this study because it is of a smaller order of magnitude than the magnitude of thermal radiation which exerts a stronger effect on the energy flux. Soret or thermal diffusion effect has been utilized for isotope separation in mixtures between gases with very light molecular weight (H_2_, He) and medium molecular weight (N_2_, air) and it was found to be of a magnitude that it cannot be neglected due to its practical applications in engineering and sciences. Soret effects due to natural convection between heated inclined plates have been investigated by Raju et al. [44]. M. G. Reddy and N. B. Reddy [45] investigated Soret and Dufour effects on steady MHD free convective flow past an infinite plate. Mohamed [46] studied unsteady MHD flow over a vertical moving porous plate with heat generation and Soret effect.

Practically, in many engineering applications, the particle adjacent to a solid surface no longer takes the velocity of the surface. The particle at the surface has a finite tangential velocity; it “slips” along the surface. This flow regime is called the slip-flow regime and this effect cannot be neglected. The fluid slippage phenomenon at the solid boundaries appear in many applications such as microchannels or nanochannels and in application where a thin film of light oils is attached to the moving plates or when the surface is coated with special coating such as thick monolayer of hydrophobic octadecyltrichlosilane, that is, lubrication of mechanical device, where a thin film of lubricant is attached to the surface slipping over one another or when the surfaces are coated with special coating to minimize the friction between them [47]. Chaudhary and Jain [48] examined the effects of radiation on the hydromagnetic free convection flow set up due to temperature as well as species concentration of an electrically conducting micropolar fluid past a vertical porous plate through porous medium in slip-flow regime. Chaudhary and Sharma [49, 50] studied the free convection flow past a vertical porous plate with variable suction in slip-flow regime. Das et al. [51] have considered the magnetohydrodynamic unsteady flow of a viscous stratified fluid through a porous medium past a porous flat moving plate in the slip flow regime with heat source. Singh and Kumar [52] presented the fluctuating heat and mass transfer on unsteady free convection flow of radiating and reacting fid past a vertical porous plate in slip flow regime using perturbation analysis. Kumar and Chand [53] have studied the effect of slip conditions and the Hall current on unsteady MHD flow of a viscoelastic fluid past an infinite vertical porous plate through porous medium. Recently, Oahimire et al. [54] investigated the effects of thermal-diffusion and thermal radiation on unsteady heat and mass transfer by free convective MHD micropolar fluid flow bounded by a semi-infinite vertical plate in a slip-flow regime under the action of transverse magnetic field with suction.

To the best of our knowledge, considerably less work has been done concerning the combined effect of Hall current and Soret effect on chemically reactive magnetomicropolar fluid flow incorporating the effect of rotation in slip flow regime in the presence of radiation and heat absorption. The results are in accordance with the physical realities which validate the correctness of our work presented here.

## 2. Mathematical Formulation of the Problem

**Figure 1:**
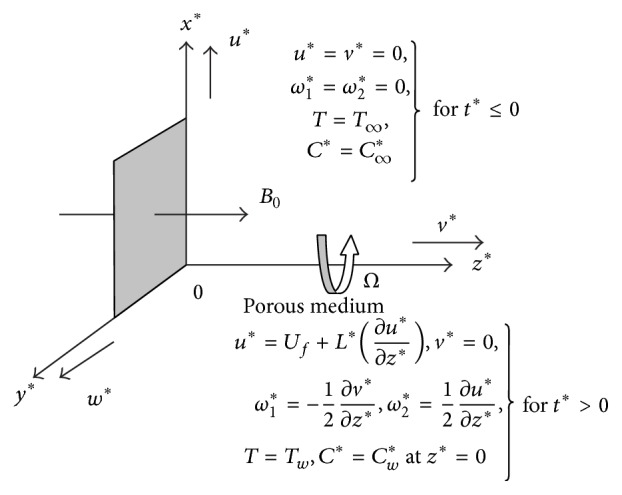
Geometry and coordinate system of the problem.

Consider an unsteady hydromagnetic flow of an incompressible, viscous, and electrically conducting micropolar fluid past an infinite vertical permeable plate embedded in a uniform porous medium in slip-flow regime and in a rotating system taking Hall current, thermal radiation, Soret effect, and chemical reaction into account. The coordinate system is chosen in such a way that *x* ^*^-axis is considered along the porous plate in vertically upward direction, *y* ^*^-axis is taken along the width of the plate, and *z* ^*^-axis normal to the plane of the plate in the fluid as shown in figure configuration (Figure 1). Since the plate is infinite in extent in *x* ^*^- and *y* ^*^- directions, hence all physical quantities will be independent of *x* ^*^ and *y* ^*^ and they are functions of *z* ^*^ and *t* ^*^ only; that is, ∂*u* ^*^/∂*x* ^*^ = ∂*u* ^*^/∂*y* ^*^ = ∂*v* ^*^/∂*x* ^*^ = ∂*v* ^*^/∂*y* ^*^ = 0, and so forth.

A magnetic field of uniform strength *B*_0_ is applied in a direction parallel to *z* ^*^-axis which is perpendicular to the flow direction. It is assumed that the induced magnetic field generated by fluid motion is negligible in comparison to the applied one. This assumption is justified because magnetic Reynolds number is very small for liquid metals and partially ionized fluids which are commonly used in industrial applications [55]. It is assumed that there is no applied or polarized voltage so the effect of polarization of fluid is negligible. This corresponds to the case where no energy is added or extracted from the fluid by electrical means. The entire system is rotating with an angular velocity *Ω* about the normal to the plate. It is assumed here that the hole size of the porous plate is significantly larger than the characteristic microscopic length scale of the porous medium. The fluid is considered to be a gray, absorbing-emitting but nonscattering medium and the Rosseland approximation is used to describe the radiative heat flux. The radiative heat flux in the *x* ^*^-direction is considered negligible in comparison with that of *z* ^*^-direction. When the strength of the magnetic field is very large, the generalized Ohm's law in the absence of electric field takes the following form:

(1) $$\begin{array}{c} \vec{J}+\frac{\omega_{e}\tau_{e}}{B_{0}}\vec{J}\times \vec{H}=\sigma \left(\mu_{e}\vec{V}\times \vec{H}+\frac{1}{{\text{en}}_{e}}\nabla P_{e}\right). \end{array}$$

Under the assumption that the electron pressure (for weakly ionized gas), the thermoelectric pressure and ion-slip conditions are negligible; now the above equation becomes

(2) $$\begin{array}{c} j_{x}=\frac{\sigma \mu_{e}H_{0}}{1+m^{2}}\left(mv-u\right),\;\;j_{z}=\frac{\sigma \mu_{e}H_{0}}{1+m^{2}}\left(mu+v\right), \end{array}$$

where *u* is the *x*-component of $\vec{V}$, *v* is the *y*-component of $\vec{V}$, and *m* (=*ω*_*e*_ *τ*_*e*_) is Hall parameter.

The suction velocity is assumed to be *w* ^*^ = −*w*_0_(1 + *εAe* ^δ^*^t^*^^), where *ε* and *εA* are small values less than unity and *w*_0_ is the scale of suction velocity which is nonzero positive constant. The negative sign indicates that the suction is towards the plate. The fluid properties are assumed to be constants except that the influence of density variation with temperature and concentration has been considered in the body-force term. There is a first-order chemical reaction between the diffusing species and the fluid.

With these foregoing assumptions, the governing equations under Boussinesq approximation can be written in a Cartesian frame of reference as follows.

*Continuity*. Consider the following:

(3) $$\begin{array}{c} \frac{\partial w^{*}}{\partial z^{*}}=0. \end{array}$$

*Linear Momentum*. Consider the following:

(4) ∂u∗∂t∗+w∗∂u∗∂z∗−2Ωv∗ =ν+νr∂2u∗∂z∗2+gβTT−T∞+gβCC∗−C∞∗  −νu∗K∗−νr∂ω2∗∂z∗+σμe2H02mv∗−u∗ρ1+m2,∂v∗∂t∗+w∗∂v∗∂z∗+2Ωu∗ =ν+νr∂2v∗∂z∗2−νu∗K∗+νr∂ω1∗∂z∗−σμe2H02mu∗+v∗ρ1+m2.

*Angular Momentum*. Consider the following:

(5) $$\begin{array}{c} \frac{\partial \omega_{1}^{*}}{\partial t^{*}}+w^{*}\frac{\partial \omega_{1}^{*}}{\partial z^{*}}=\frac{\Lambda }{\rho j}\frac{\partial^{2}\omega_{1}^{*}}{\partial z^{*2}},\\ \frac{\partial \omega_{2}^{*}}{\partial t^{*}}+w^{*}\frac{\partial \omega_{2}^{*}}{\partial z^{*}}=\frac{\Lambda }{\rho j}\frac{\partial^{2}\omega_{2}^{*}}{\partial z^{*2}}. \end{array}$$

*Energy*. Consider the following:

(6) ∂T∂t∗+w∗∂T∂z∗=kρCp∂2T∂z∗2−Q∗ρCpT−T∞−1ρCp∂qr∂z∗.

*Mass Transfer*. Consider the following:

(7) ∂C∗∂t∗+w∗∂C∗∂z∗=Dm∂2C∗∂z∗2+DmKtTm∂2T∗∂z∗2−RCC∗−C∞∗.

The initial and boundary conditions suggested by the physics of the problem are

(8) u∗=v∗=0,  ω1∗=ω2∗=0,T=T∞,  C∗=C∞∗kkkkkkkkkkkkkkikkfor  t∗≤0,

(9) u∗=Ur+L∗∂u∗∂z∗,  v∗=0,ω1∗=−12∂v∗∂z∗,  ω2∗=12∂u∗∂z∗,T=Tw,  C∗=Cw∗ikkkkkkkkkkkkkkkkkkkat  z∗=0u∗⟶0,  v∗⟶0,  ω1∗⟶0,ω2∗⟶0,  T⟶T∞,  C∗⟶C∞∗ikkkkkkkkkkkkkkkkkkkkkias  z∗⟶∞ikkkkkkkkkkkkkkkkkkkkkkikfor  t∗>0.

The boundary condition for microrotation components *ω*_1_^*^ and *ω*_2_^*^ describes its relationship with the surface stress. In the above boundary condition (9) the plate is in uniform motion and subjected to variable suction and slip boundary condition. In the parameter *L* ^*^ = ((2 − *m*_1_)/*m*_1_)*L*, *L* is the molecular mean free path and *m*_1_ is the tangential momentum accommodation coefficient. All the physical variables are given in nomenclature.

Integration of continuity equation (3) for variable suction velocity normal to the plate gives

(10) $$\begin{array}{c} w^{*}=-w_{0}\left(1+\varepsilon Ae^{\delta^{*}t^{*}}\right), \end{array}$$

where *w*_0_ represents the normal velocity at the plate which is positive for suction and negative for blowing. The radiative heat flux term by using Rosseland approximation is given by

(11) $$\begin{array}{c} q_{r}=-\frac{4\sigma^{*}}{3a_{R}}\frac{\partial T^{4}}{\partial z^{*}}. \end{array}$$

We assume that the temperature differences within the flow are such that *T* ^4^ may be expressed as a linear function of the temperature *T*. This is accomplished by expanding *T* ^4^ in a Taylor series about *T*_*∞*_ and, neglecting higher-order terms, we have

(12) $$\begin{array}{c} T^{4}≃4T_{\infty }^{3}T-3T_{\infty }^{4}. \end{array}$$

By using (11) and (12), (6) gives

(13) ∂T∂t∗+w∗∂T∂z∗=kρCp∂2T∂z∗2−Q∗ρCpT−T∞+16σ∗T∞33ρCpaR∂2T∂z∗2.

Proceeding with analysis, we introduce the following dimensionless variables:

(14) $$\begin{array}{c} u=\frac{u^{*}}{U_{r}},\;\;v=\frac{v^{*}}{U_{r}},\;\;z=\frac{z^{*}U_{r}}{\nu },\\ t=\frac{t^{*}U_{r}^{2}}{\nu },\;\;\delta =\frac{\delta^{*}\nu }{U_{r}^{2}},\;\;\omega_{1}=\frac{\omega_{1}^{*}\nu }{U_{r}^{2}},\;\;\omega_{2}=\frac{\omega_{2}^{*}\nu }{U_{r}^{2}},\\ \text{Gr}=\frac{\nu g\beta_{T}\left(T_{w}-T_{\infty }\right)}{U_{r}^{3}},\;\;\text{Gc}=\frac{\nu g\beta_{C}\left(C_{w}^{*}-C_{\infty }^{*}\right)}{U_{r}^{3}},\\ R=\frac{2\Omega \nu }{U_{r}^{2}},\;\;S=\frac{w_{0}}{U_{r}},\;\;\Delta =\frac{\nu_{r}}{\nu },\\ \theta =\frac{T-T_{\infty }}{T_{w}-T_{\infty }},\;\;C=\frac{C^{*}-C_{\infty }^{*}}{C_{w}^{*}-C_{\infty }^{*}},\;\;K=\frac{K^{*}U_{r}^{2}}{\nu^{2}},\\ M=\frac{\mu_{e}H_{0}}{U_{r}}\sqrt{\frac{\sigma \nu }{\rho }},\;\;\lambda =\frac{\Lambda }{\mu j},\;\;\mathrm{Pr}=\frac{\mu C_{p}}{k},\\ \text{Sc}=\frac{\nu }{D_{m}},\;\;F=\frac{4T_{\infty }^{3}\sigma^{*}}{ka_{R}},\;\;Q=\frac{Q^{*}\nu^{2}}{U_{r}^{2}k},\\ \text{Sr}=\frac{D_{m}K_{t}\left(T_{W}-T_{\infty }\right)}{T_{m}\left(C_{W}^{*}-C_{\infty }^{*}\right)\nu },\;\;\alpha =\frac{R_{C}\nu }{U_{r}^{2}},\;\;h=\frac{L^{*}U_{r}}{\nu }. \end{array}$$

In view of (14), the governing equations (4)–(7) and (13) reduce to the following dimensionless form:

(15) ∂u∂t−S1+εAeδt∂u∂z−Rv =1+Δ∂2u∂z2+Grθ+Gmϕ−M21+m2+1ku  −Δ∂ω2∂z+mM21+m2v,

(16) ∂v∂t−S1+εAeδt∂v∂z+Ru =1+Δ∂2v∂z2−M21+m2+1kv+Δ∂ω1∂z−mM21+m2u,

(17) $$\begin{array}{c} \frac{\partial \omega_{1}}{\partial t}-S\left(1+\varepsilon Ae^{\delta t}\right)\frac{\partial \omega_{1}}{\partial z}=\lambda \frac{\partial^{2}\omega_{1}}{\partial z^{2}}, \end{array}$$

(18) $$\begin{array}{c} \frac{\partial \omega_{2}}{\partial t}-S\left(1+\varepsilon Ae^{\delta t}\right)\frac{\partial \omega_{2}}{\partial z}=\lambda \frac{\partial^{2}\omega_{2}}{\partial z^{2}}, \end{array}$$

(19) $$\begin{array}{c} \frac{\partial \theta }{\partial t}-S\left(1+\varepsilon Ae^{\delta t}\right)\frac{\partial \theta }{\partial z}=\frac{1}{\mathrm{Pr}}\left(1+\frac{4F}{3}\right)\frac{\partial^{2}\theta }{\partial z^{2}}-\frac{Q}{\mathrm{Pr}}\theta , \end{array}$$

(20) $$\begin{array}{c} \frac{\partial C}{\partial t}-S\left(1+\varepsilon Ae^{\delta t}\right)\frac{\partial C}{\partial z}=\frac{1}{\text{Sc}}\frac{\partial^{2}C}{\partial z^{2}}+\text{Sr}\frac{\partial^{2}C}{\partial z^{2}}-\alpha C. \end{array}$$

The boundary conditions (8)-(9) in view of (14) are then given by the following dimensionless form:

(21) u=v=0,  ω1=ω2=0,  θ=0,  C=0kkkkkkkkkkkkkkkkkkkkkkkkkkkkkfor  t≤0u=1+h∂u∂z,  v=0,  ω1=−12∂v∂z,ω2=12∂u∂z,  θ=1,  C=1kkkkkkkkkkkkkkkkkkkkkkkkkat  z=0u⟶0,  ω1⟶0,  ω2⟶0,θ⟶0,  C⟶0kkkkkkkkkkkkkkkkkkkkas  z⟶∞kkkkkkkkkkkkkkkkkkkkkkkfor  t>0.

To simplify (15)–(18), we substitute the fluid velocity and angular velocity in the complex form as *V* = *u* + *iv*,  *ω* = *ω*_1_ + *iω*_2_ and we get

(22) ∂V∂t−S1+εAeδt∂V∂z+iRV =1+Δ∂2V∂z2+Grθ+Gmϕ−M21+m2+1kV  −iΔ∂ω∂z−imM21+m2V,∂ω∂t−S1+εAeδt∂ω∂z=λ∂2ω∂z2.

The associated boundary conditions (21) become

(23) V=0,  ω=0,  θ=0,  C=0kkkkkkkkkkkkikkkkkkkkkkfor  t≤0V=1+h∂u∂z,  ω=i2∂V∂z,  θ=1,  C=1kkkkkkkkkkkkkkkkkkkkkkkkkkkkkkkiat  z=0V⟶0,  ω⟶0,  θ⟶0,  C⟶0kkkkkkkkkkkkkkkkkkikkkkkkkkkkas  z⟶∞kkkkkkkkkkkkkkkkkkkkkkkkkkkkkkkfor  t>0.

## 3. Analytical Solution of the Problem

In order to reduce the above system of partial differential equations to a system of ordinary differential equations in dimensionless form, we represent the translational velocity *V*, microrotation velocity *ω*, temperature *θ*, and concentration *C* as

(24) $$\begin{array}{c} V\left(z,t\right)=V_{0}\left(z\right)+\varepsilon e^{\delta t}V_{1}\left(z\right)+O\left(\varepsilon^{2}\right),\\ \omega \left(z,t\right)=\omega_{0}\left(z\right)+\varepsilon e^{\delta t}\omega_{1}\left(z\right)+O\left(\varepsilon^{2}\right),\\ \theta \left(z,t\right)=\theta_{0}\left(z\right)+\varepsilon e^{\delta t}\theta_{1}\left(z\right)+O\left(\varepsilon^{2}\right),\\ C\left(z,t\right)=C_{0}\left(z\right)+\varepsilon e^{\delta t}C_{1}\left(z\right)+O\left(\varepsilon^{2}\right). \end{array}$$

By substituting the above equations (24) into (19), (20), (22)-(23) and equating the harmonic and nonharmonic terms and neglecting the higher-order terms of *O*(*ε* ^2^), we obtain the following pairs of equations for (*V*_0_, *ω*_0_, *θ*_0_, *C*_0_) and (*V*_1_, *ω*_1_, *θ*_1_, *C*_1_).

Zero-order equations are:

(25) $$\begin{array}{c} \left(1+\Delta \right)V_{0}^{\prime \prime }+SV_{0}^{\prime }-a_{1}V_{0}+\text{Gr}\theta_{0}+\text{Gm}C_{0}+i\Delta \omega_{0}^{\prime }=0,\\ \lambda \omega_{0}^{\prime \prime }+S\omega_{0}^{\prime }=0,\\ \left(3+4F\right)\theta_{0}^{\prime \prime }+3\mathrm{Pr}S\theta_{0}^{\prime }-3Q\theta_{0}=0,\\ C_{0}^{\prime \prime }+S\text{Sc}C_{0}^{\prime }-\alpha \text{Sc}C_{0}=-\text{Sr}\theta_{0}^{\prime \prime }. \end{array}$$

First-order equations are:

(26) 1+ΔV1′′+SV1′−a2V1+Grθ1+GmC1  +AV0′+iΔω1′=0,λω1′′+Sω1′−δω1=−SAω0′,3+4Fθ1′′+3Pr⁡Sθ1′−3Q+Pr⁡δθ1 =−3Pr⁡SAθ0′,C1′′+SScC1′−Scα+δC1=−SScAC0′−SrScθ1′′.

The prime denotes differentiation with respect to *y*.

The corresponding boundary conditions can be written as

(27) V0=1+h∂V0∂z,  V1=h∂V1∂z,ω0=i2∂V0∂z,  ω1=i2∂V1∂z,θ0=1, θ1=0, C0=1, C1=0 at  z=0V0⟶0,  V1⟶0,  ω0⟶0,ω1⟶0,  θ0⟶0,  θ1⟶0,C0⟶0,  C1⟶0kkkkkkkkkkkas  z⟶∞.

Solving (25)-(26) satisfying the boundary conditions (27) we obtain the expression for translational velocity *V*, microrotation velocity *ω*, temperature *θ*, and concentration *C* as

(28) Vz,t=B11e−r5z+B8e−r1z+B9e−r3z+B10e−S/λz+εeδtB20e−r7z+B13e−r1z+B17e−r5z+B18e−S/λz+B19e−r6z     +B14e−r2z+B15e−r4z+B16e−r3z     + B17e−r5z+B18e−S/λz+B19e−r6z,

(29) $$\begin{array}{c} \omega \left(z,t\right)=D_{1}e^{-\left(S/\lambda \right)z}+\varepsilon e^{\delta t}\left\{\left.D_{2}e^{-r_{6}z}+B_{12}e^{-\left(S/\lambda \right)z}\right\}\right., \end{array}$$

(30) $$\begin{array}{c} \theta \left(z,t\right)=e^{-r_{1}z}+\varepsilon e^{\delta t}\left\{B_{1}\left(e^{-r_{1}z}-e^{-r_{2}z}\right)\right\}, \end{array}$$

(31) Cz,t=B3e−r3z+B2e−r1z+εeδtB4e−r3z+B5e−r1z       + B6e−r2z+B7e−r4z.

The exponential indices and the coefficients appearing in (28)–(31) are given in the appendix.

In technological applications, the wall shear stress, the wall couple stress, and the heat and mass transfer rate are often of great interest. Skin friction is caused by viscous drag in the boundary layer around the plate. The skin friction coefficient (*C*_*f*_) at the wall in dimensionless form is given by

(32)  Cf=τw∗z∗=0ρUr2=1+Δ1+i2∂V∂zz=0

(33)  =−1+Δ1+i2    ×B11r5+B8r1+B9r3+B10Sλ+B17r5+B18Sλ+B19r6      +εeδtB20r7+B13r1+B17r5+B18Sλ+B19r6            +B14r2+B15r4+B16r3            + B17r5+B18Sλ+B19r6.

The couple stress coefficient (*C*_*m*_) at the plate is defined by

(34) $$\begin{array}{c} M_{w}={\left.\Lambda \frac{\partial \omega^{*}}{\partial z^{*}}\right|}_{z^{*}=0} \end{array}$$

and in the dimensionless form it is given by

(35) Cm=MwμjUr=1+Δ2∂ω∂zz=0=1+Δ2∂ω1∂zz=0+i∂ω2∂zz=0=−1+Δ2D1Sλ+εeδtD2r6+B12Sλ.

Knowing the temperature field, it is interesting to study the effect of the free convection and thermal radiation on the rate of heat transfer and this is given by

(36) $$\begin{array}{c} q_{w}^{*}=-k\frac{\partial T}{\partial z^{*}}-\frac{4\sigma^{*}}{3a_{R}}{\left.\left(\frac{\partial T^{4}}{\partial z^{*}}\right)\right|}_{z^{*}=0}. \end{array}$$

Using *T* ^4^≃4*T*_*∞*_^3^ *T* − 3*T*_*∞*_^4^ the above equation becomes

(37) $$\begin{array}{c} q_{w}^{*}=-k\left(T_{w}-T_{\infty }\right)\frac{U_{r}}{\nu }\left(1+\frac{4F}{3}\right){\left.\frac{\partial \theta }{\partial z}\right|}_{z=0}. \end{array}$$

The rate of heat transfer between the fluid and the plate is studied in terms of nondimensional Nusselt number, which is given by

(38) Nu=xqw∗kTw−T∞=−Rex1+4F3∂θ∂zz=0,

where Re_*x*_ = *U*_*r*_ *x*/*ν* is the local Reynolds number

(39) NuRex−1=1+4F3∂θ∂zz=0=1+4F3r1+εeδtB1r1−r2.

The definitions of the local mass flux and the local Sherwood number are, respectively, given by

(40) jw=−Dm∂C∗∂z∗z∗=0,Shx=jwxDmCw∗−C∞∗=−Rex∂C∂zz=0,ShxRex−1=∂C∂zz=0=B3r3+B2r1+εeδtB4r3+B5r1+B6r2+B7r4.

## 4. Results and Discussion

In the preceding sections, the governing equations along with the boundary conditions are solved analytically employing the perturbation techniques. The effects of main controlling parameters as they appear in the governing equations are discussed on the temperature *θ*, concentration *C*, translational velocity *V*, microrotation *ω*, skin-friction *C*_*f*_, Nusselt number, and Sherwood number. In order to get a physical insight of the problem the above physical quantities are compiled numerically and displayed graphically. In the entire calculations we have chosen *ε* = 0.01, *δ* = 0.1, *t* = 1 and *A* = 1 while Pr, *S*, *F*, *Q*, Sr, Sc, *M*, *m*, Gr, Gm, *R*, *h*, *K*, Δ, and *λ* are varied over the range which are listed in the figure legends.

**Figure 2:**
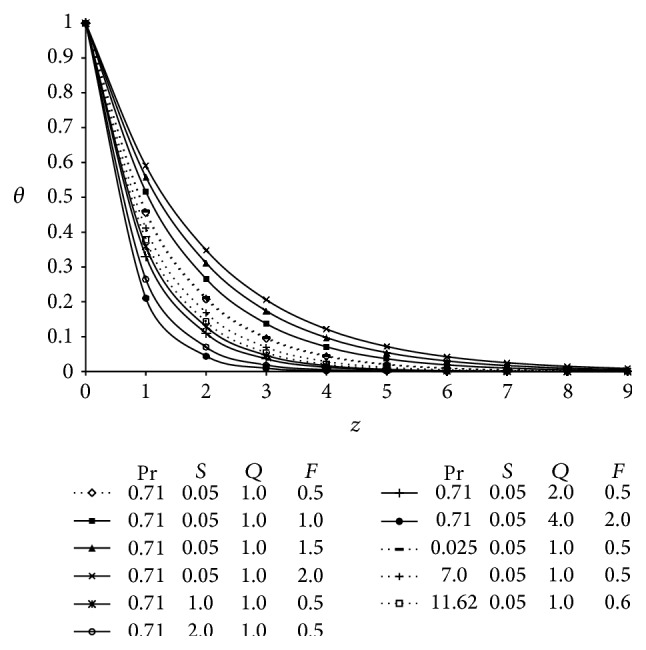
Temperature profiles for different values of Prandtl number (Pr), suction parameter (*S*), heat absorption parameter (*Q*), and radiation parameter (*F*) taking *A* = 3, *t* = 1, and *δ* = 0.1.

The numerical values of fluid temperature *θ* computed from the analytical solutions (29) are illustrated graphically versus boundary layer coordinate *z* in Figure 2 for various values of Prandtl number (Pr), suction parameter (*S*), heat absorption parameter (*Q*), and radiation parameter (*F*). The values of Prandtl number are chosen as Pr = 0.71, 0.025, and 7.0 which physically correspond to air, mercury, and water at 25° temperature and one atmospheric pressure. Pr = 11.62 correspond to water at 4°C. It is inferred that the temperature falls more rapidly for water in comparison to air which is physically true thus the thermal boundary layer falls quickly for large value of Prandtl number. The thickness of thermal boundary layer is greatest for Pr = 0.025 (mercury) than for Pr = 0.71 (air), thereafter for Pr = 7 (water) and finally the lowest for Pr = 11.62 (water at 4°C); that is, an increase in Prandtl number results in a decrease of temperature. The reason underlying such a behavior is that Pr signifies the relative effects of viscosity to thermal conductivity and smaller values of Prandtl number possess high thermal conductivity and therefore heat is able to diffuse away from the surface faster than at higher values of Pr. This results in the reduction of thermal boundary layer thickness. The fluid temperature *θ* also decreases with an increase of Heat absorption parameter (*Q*) and suction parameter (*S*). The temperature decreases with an increase in the heat absorption parameter because when heat is absorbed the buoyancy forces decrease the temperature profiles. The effect of thermal radiation parameter (*F*) is to enhance the fluid temperature throughout the boundary layer region. This is consistent with the fact that thermal radiation provides an additional means to diffuse energy because thermal radiation parameter *F* = 4*T*_*∞*_^3^ *σ* ^*^/*ka*_*R*_ and therefore an increase in *F* implies a decrease in Rosseland mean absorption coefficient *a*_*R*_ for fixed values of *T*_*∞*_ and *k*. Thus it is pointed out that radiation should be minimized to have the cooling process at a faster rate. The temperature profiles attain their maximum value at the wall and decrease exponentially with *z* and finally tend to zero as *z* → *∞*. Hence the accuracy is checked and it validates that the analytical results for temperature is correct.

**Figure 3:**
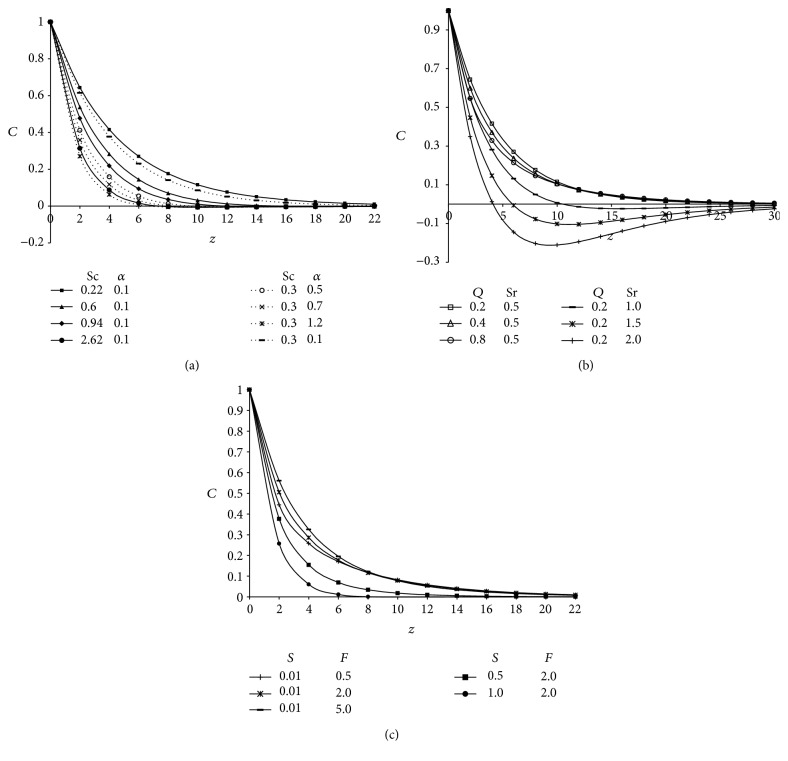
(a) Concentration profiles showing the variation for Schmidt number (Sc) and chemical reaction parameter (*α*) taking Pr = 0.71, *S* = 0.01, *F* = 2, *Q* = 1, *δ* = 0.1, *t* = 1, and *A* = 3. (b) Concentration profiles showing the variation for heat generation parameter (*Q*) and Soret number (Sr) taking Pr = 0.71, *S* = 0.01, *F* = 2, Sc = 0.22, *δ* = 0.1, *t* = 1, and *A* = 3. (c) Concentration profiles showing the variation for suction parameter (*S*) and radiation parameter (*F*) taking Pr = 0.71, Sr = 0.5, Sc = 0.3, *Q* = 1, *δ* = 0.1, *t* = 1, and *A* = 3.

Graphical results of concentration profiles *C* for different values of Schmidt number (Sc) and chemical reaction parameter (*α*) are displayed in Figure 3(a). The values of Schmidt number are chosen to represent the most common diffusing chemical species which are of interest and they are Sc = 0.22 (hydrogen), Sc = 0.3 (helium), Sc = 0.6 (water vapor), Sc = 0.94 (carbon dioxide) and Sc = 2.62 (propylbenzene) at 25°C temperature and one atmospheric pressure. A comparison of curves in the figure show the concentration distribution decreases at all points in the flow field with an increase in Schmidt number because smaller values of Sc are equivalent to increasing chemical molecular diffusivity (*D*). This implies mass diffusion tends to enhance species concentration. This shows that the heavier diffusing species have a greater retarding effect on the concentration distribution. Furthermore, it is interesting to note that concentration profiles fall slowly and steadily for hydrogen (Sc = 0.22) and helium (Sc = 0.30) but falls very rapidly for water vapor (Sc = 0.6) and propylbenzene (Sc = 2.62). Physically this is true because of the fact that the water vapor can be used for maintaining normal concentration field whereas hydrogen can be used for maintaining effective concentration field. Similar effects are seen in the case when chemical reaction parameter (*α*) is increased. Further, this figure clearly demonstrates that the concentration profiles decrease rapidly when chemical reaction parameter is increased this is due to the fact that boundary layer decreases with an increase in the value of *α* in this system, results in the consumption of the chemical and hence result in decreasing concentration profile. Thus the diffusion rates can be tremendously altered by chemical reaction.

The effects of heat absorption parameter (*Q*) and Soret number (Sr) on concentration profiles across the boundary layer are displayed in Figure 3(b). The results show that concentration boundary layer suppresses with an increase in heat absorption parameter and Soret number. The profiles fall rapidly with an increase of Soret number and thereafter increase and tend to zero as *z* → *∞*.  Figure 3(c) is plotted to show the effects of radiation parameter (*F*) and Suction parameter (*S*) on the species concentration profiles. It is revealed that the presence of Suction parameter diminishes the concentration distribution whereas reverse phenomena are observed with increasing values of radiation parameter. In Figures 3(a)–3(c) the concentration profiles attain their maximum value at the wall and decrease exponentially with *z* and finally tend to zero as *z* → *∞*. Hence it is found to be in good agreement with boundary condition given in (23). Moreover these figures provide a check of our analytical solution for the concentration field.

**Figure 4:**
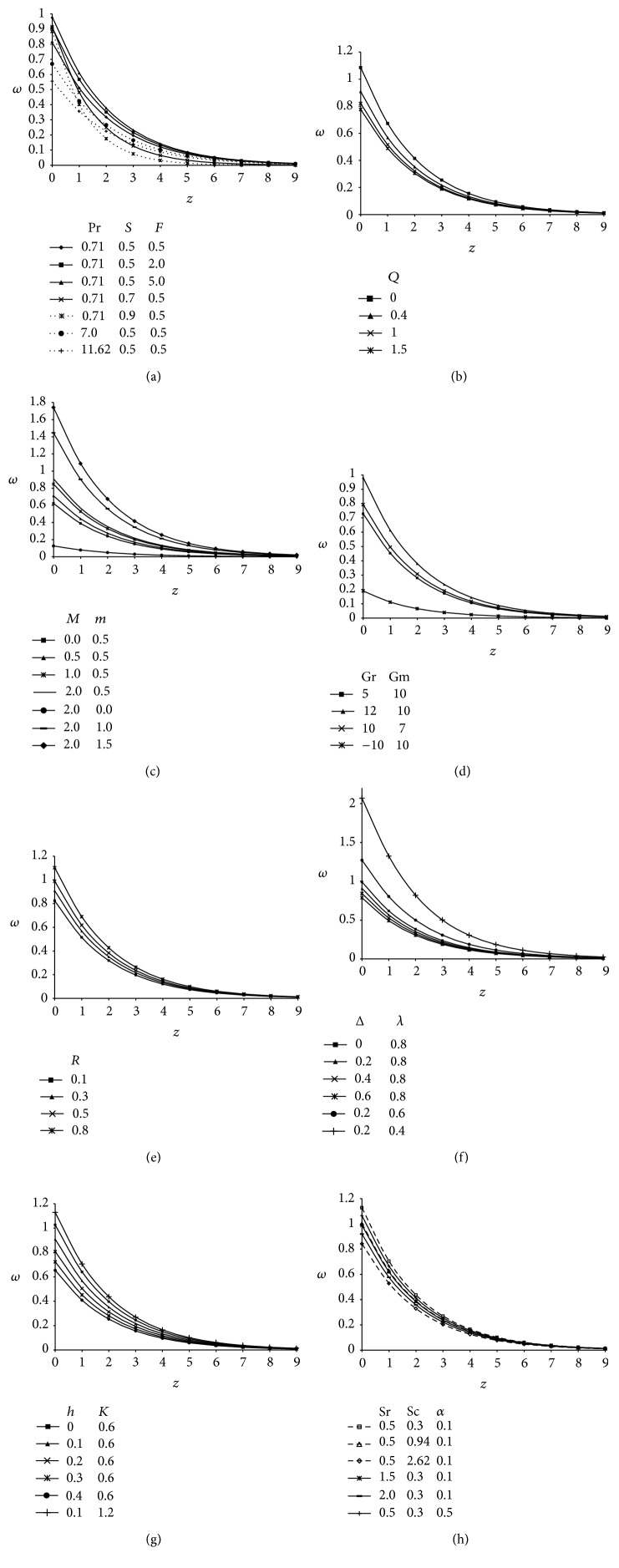
(a) Microrotation profiles showing the variation of Prandtl number (Pr), suction parameter (*S*), and radiation parameter (*F*) taking *Q* = 0.4, *α* = 0.1, *δ* = 0.1, *t* = 1, *A* = 3, Sr = 0.5, Sc = 0.3, *M* = 2, *m* = 0.5, Gr = 10, Gm = 10, *R* = 0.3, *h* = 0.1, *K* = 0.6, Δ = 0.2, and *λ* = 0.8. (b) Microrotation profiles showing the variation of heat absorption parameter (*Q*) taking Pr = 0.71, *S* = 0.5, *F* = 2, *α* = 0.1, *δ* = 0.1, *t* = 1, *A* = 3, Sr = 0.5, Sc = 0.3, *M* = 2, *m* = 0.5, Gr = 10, Gm = 10, *R* = 0.3, *h* = 0.1, *K* = 0.6, Δ = 0.2, and *λ* = 0.8. (c) Microrotation profiles showing the variation of magnetic parameter (*M*) and Hall parameter (*m*) taking Pr = 0.71, *S* = 0.5, *F* = 2, *Q* = 0.4, *α* = 0.1, *δ* = 0.1, *t* = 1, *A* = 3, Sr = 0.5, Sc = 0.3, Gr = 10, Gm = 10, *R* = 0.3, *h* = 0.1, *K* = 0.6, Δ = 0.2, and *λ* = 0.8. (d) Microrotation profiles showing the variation of Grashof number (Gr) and modified Grashof number (Gm) taking Pr = 0.71, *S* = 0.5, *F* = 2, *Q* = 0.4, *α* = 0.1, *δ* = 0.1, *t* = 1, *A* = 3, Sr = 0.5, Sc = 0.3, *M* = 2, *m* = 0.5, *R* = 0.3, *h* = 0.1, *K* = 0.6, Δ = 0.2, and *λ* = 0.8. (e) Microrotation profiles showing the variation of rotational parameter (*R*) taking Pr = 0.71, *S* = 0.5, *F* = 2, *Q* = 0.4, *α* = 0.1, *δ* = 0.1, *t* = 1, *A* = 3, Sr = 0.5, Sc = 0.3, *M* = 2, *m* = 0.5, Gr = 10, Gm = 10, *h* = 0.1, *K* = 0.6, Δ = 0.2, and *λ* = 0.8. (f) Microrotation profiles showing the variation of viscosity ratio (Δ) and material parameter (*λ*) taking Pr = 0.71, *S* = 0.5, *F* = 2, *Q* = 0.4, *α* = 0.1, *δ* = 0.1, *t* = 1, *A* = 3, Sr = 0.5, Sc = 0.3, *M* = 2, *m* = 0.5, Gr = 10, Gm = 10, *R* = 0.3, *h* = 0.1, and *K* = 0.6. (g) Microrotation profiles showing the variation of slip parameter (*h*) and permeability parameter (*K*) taking Pr = 0.71, *S* = 0.5, *F* = 2, *Q* = 0.4, *α* = 0.1, *δ* = 0.1, *t* = 1, *A* = 3, Sr = 0.5, Sc = 0.3, *M* = 2, *m* = 0.5, Gr = 10, Gm = 10, *R* = 0.3, Δ = 0.2, and *λ* = 0.8. (h) Microrotation profiles showing the variation of Soret parameter (Sr), Schmidt number (Sc), and chemical reaction parameter (*α*) taking Pr = 0.71, *S* = 0.5, *F* = 2, *Q* = 0.4, *δ* = 0.1, *t* = 1, *A* = 3, *M* = 2, *m* = 0.5, Gr = 10, Gm = 10, *R* = 0.3, *h* = 0.1, *K* = 0.6, and Δ = 0.2.

The microrotation profiles (*ω*) against span wise coordinate *z* incorporating the effect of various parameters influencing the flow field are demonstrated in Figures 4(a)–4(h). It is revealed from Figures 4(a)–4(h) that these profiles attain a distinctive maximum value near surface of the plate and decrease properly on increasing boundary layer coordinate *z* to approach free stream value.  Figure 4(a) shows the influence of Prandtl number (Pr), Suction parameter (*S*) and radiation parameter (*F*) on microrotation profiles. It is noticed that microrotation profiles (*ω*) decrease on increasing Pr. Physically, it is true due to the fact that an increase in Prandtl number increase the viscosity of the fluid, so the fluid becomes thick and consequently leads to a decrease in velocity. This figure further indicates that the microrotation profiles decrease with an increase in suction parameter (*S*) because sucking decelerates the fluid particles through the porous wall and hence reduce the growth of the fluid boundary layer as well as thermal and concentration boundary layers. Indicating the usual fact that suction stabilizes the boundary layer growth. These profiles enhances with an increase in radiation parameter (*F*). This is because when the intensity of heat generated through thermal radiation increases, the bond holding the components of the fluid particle is easily broken and the fluid velocity will increase.

From Figure 4(b) it is perceived that microrotation profiles decrease with an increase in heat absorption parameter (*Q*).  Figure 4(c) elucidates the influence of magnetic parameter (*M*) and Hall parameter (*m*) on microrotation profiles (*ω*); it is clear from these curves that these profiles increase when magnetic parameter and Hall current parameter are increased. The profiles corresponding to *m* = 0 reveals that microelements close to the wall are unable to rotate; hence, *ω* is very small.  Figure 4(d) demonstrates the effect of thermal and concentration buoyancy forces, that is, Grashof number (Gr) and modified Grashof number (Gm) on the microrotation profiles. Here the negative value of Grashof number (Gr < 0), physically, corresponds to heating of the plate while the positive value (Gr > 0) represents cooling of the plate. Hence, it is observed from the comparison of the curves that an increase in thermal Grashof number leads to an increase in the velocity due to an enhancement in buoyancy forces. Gr signifies the relative strength of thermal buoyancy force to viscous hydrodynamic force. An increase in Grashof number indicates small viscous effects in the momentum equation and consequently causes an increase in the velocity profiles. Furthermore, the comparison of the curves illustrates that velocity increases with increasing Gm. The modified Grashof number (Gm) represents the relative strength of concentration buoyancy forces to viscous hydrodynamic force. As expected, the fluid velocity increases and the peak value is more distinctive due to an increase in the species buoyancy force. The profiles attain a maximum value near the wall and then decrease rapidly to approach the free stream value. Hence we are confident at the accuracy of our solution given by (30).

For various values of rotational parameter (*R*), the profiles of microrotation across the boundary layer are shown in Figure 4(e). It is perceived that the rotation tend to decrease the microrotation profiles.  Figure 4(f) presents the effect of viscosity ratio (Δ) and material parameter (*λ*) on *ω*. The magnitude of microrotation is greater for a Newtonian fluid (Δ = 0) with given parameters as compared with micropolar fluids (Δ ≠ 0). Also, it is observed that the magnitude of microrotation profiles decrease with an increase in material parameter (*λ*) and viscosity ratio (Δ).

Rarefaction effects that give rise to slip flow become significant when the molecular mean free path is comparable to characteristic length of the system. The microrotation profiles presented in Figure 4(g) incorporate the influence of rarefaction parameter (*h*) and permeability parameter (*K*). It is noticed that an increase in the value of rarefaction parameter decreases the magnitude of microrotation profiles while the comparison of curves for different values of permeability parameter (*K*) reflects that profiles increase with increasing values of *K*. A similar behavior is also expected because when we increase the permeability it increases the size of the pores inside the porous medium due to which the drag force decreases and hence the magnitude of microrotation profiles increases.

Microrotation profiles showing the variation of Soret parameter (Sr), Schmidt number (Sc), and generative chemical reaction (*α*) are presented in Figure 4(h). It is analyzed that the influence of Sr, Sc, and *α* is to reduce the magnitude of microrotation profiles. Comparison of the curves in this figure indicate that the magnitude of microrotation profiles is the greatest for helium (He: Sc = 0.3) and then for carbon dioxide (CO_2_: Sc = 0.94) and the lowest for propylbenzene (C_9_H_10_: Sc = 2.62). Physically it is justified because, for large value of Schmidt number, the fluid becomes denser. This figure also displays the fact that these profiles decrease during the destructive reaction (*α* > 0).

**Figure 5:**
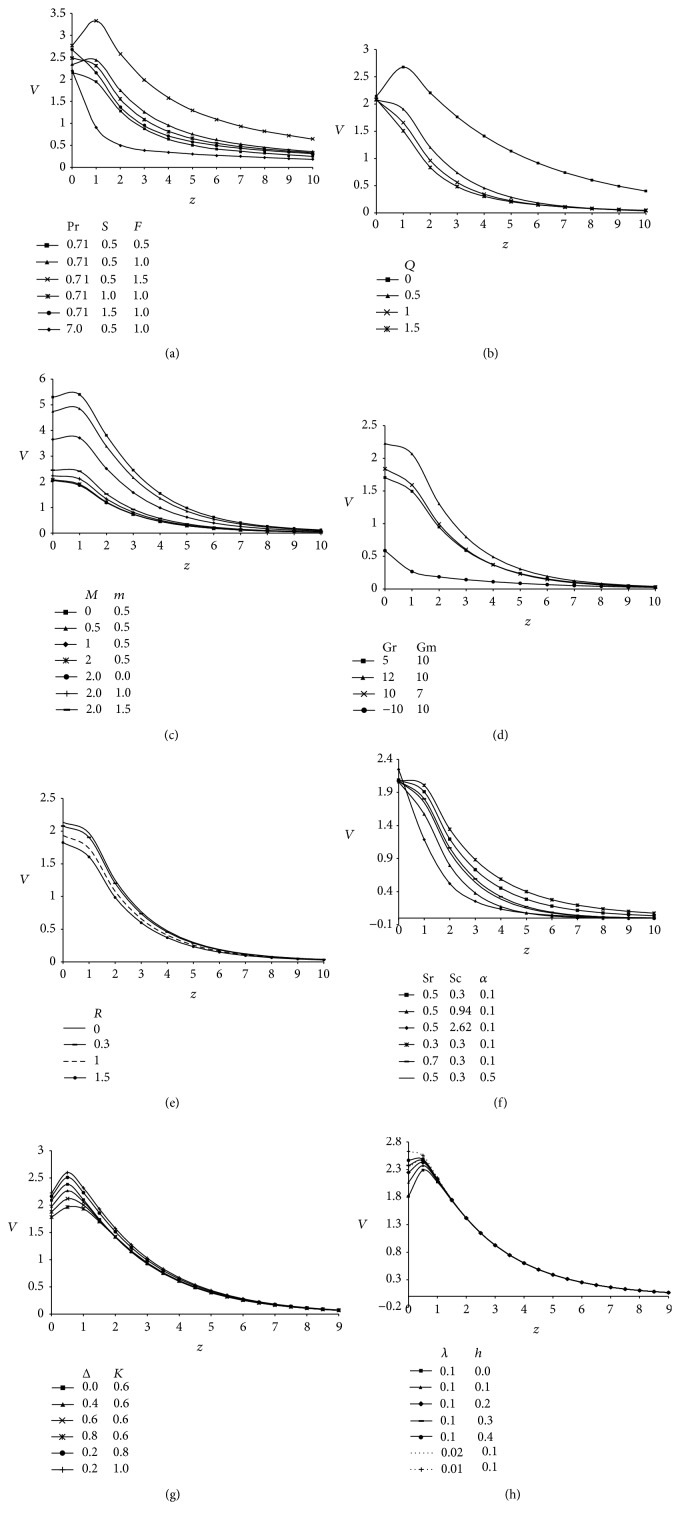
(a) Velocity profiles showing the variation of Prandtl number (Pr), suction parameter (*S*), and radiation parameter (*F*) taking *Q* = 0.5, *α* = 0.1, *δ* = 0.1, *t* = 1, *A* = 3, Sr = 0.5, Sc = 0.3, *M* = 2, *m* = 0.5, Gr = 10, Gm = 10, *R* = 0.3, *h* = 0.1, *K* = 0.6, Δ = 0.2, and *λ* = 0.8. (b) Velocity profiles showing the variation of heat absorption parameter (*Q*) taking Pr = 0.71, *S* = 0.5, *F* = 1, *α* = 0.1, *δ* = 0.1, *t* = 1, *A* = 3, Sr = 0.5, Sc = 0.3, *M* = 2, *m* = 0.5, Gr = 10, Gm = 10, *R* = 0.3, *h* = 0.1, *K* = 0.6, Δ = 0.2, and *λ* = 0.8. (c) Velocity profiles showing the variation of magnetic parameter (*M*) and Hall parameter (*m*) taking Pr = 0.71, *S* = 0.5, *F* = 1, *Q* = 0.5, *α* = 0.1, *δ* = 0.1, *t* = 1, *A* = 3, Sr = 0.5, Sc = 0.3, Gr = 10, Gm = 10, *R* = 0.3, *h* = 0.1, *K* = 0.6, Δ = 0.2, and *λ* = 0.8. (d) Velocity profiles showing the variation of Grashof number (Gr) and modified Grashof number (Gm) taking Pr = 0.71, *S* = 0.5, *F* = 1, *Q* = 0.5, *α* = 0.1, *δ* = 0.1, *t* = 1, *A* = 3, Sr = 0.5, Sc = 0.3, *M* = 2, *m* = 0.5, *R* = 0.3, *h* = 0.1, *K* = 0.6, Δ = 0.2, and *λ* = 0.8. (e) Velocity profiles showing the variation of rotational parameter (*R*) taking Pr = 0.71, *S* = 0.5, *F* = 1, *Q* = 0.5, *α* = 0.1, *δ* = 0.1, *t* = 1, *A* = 3, Sr = 0.5, Sc = 0.3, *M* = 2, *m* = 0.5, Gr = 10, Gm = 10, *h* = 0.1, *K* = 0.6, Δ = 0.2, and *λ* = 0.8. (f) Velocity profiles showing the variation of Soret parameter (Sr), Schmidt number (Sc), and chemical reaction parameter (*α*) taking Pr = 0.71, *S* = 0.5, *F* = 1, *Q* = 0.5, *δ* = 0.1, *t* = 1, *A* = 3, *M* = 2, *m* = 0.5, Gr = 10, Gm = 10, *R* = 0.3, *h* = 0.1, *K* = 0.6, Δ = 0.2, and *λ* = 0.8. (g) Velocity profiles showing the variation of viscosity ratio (Δ) and permeability parameter (*K*) taking Pr = 0.71, *S* = 0.5, *F* = 2, *Q* = 0.5, *α* = 0.1, *δ* = 0.1, *t* = 1, *A* = 3, Sr = 0.5, Sc = 0.3, *M* = 2, *m* = 0.5, Gr = 10, Gm = 10, *R* = 0.3, *h* = 0.1, and *λ* = 0.1. (h) Velocity profiles showing the variation of slip parameter (*h*) and material parameter (*λ*) taking Pr = 0.71, *S* = 0.5, *F* = 2, *Q* = 0.5, *α* = 0.1, *δ* = 0.1, *t* = 1, *A* = 3, Sr = 0.5, Sc = 0.3, *M* = 2, *m* = 0.5, Gr = 10, Gm = 10, *R* = 0.3, Δ = 0.2, and *K* = 0.6.

Figures 5(a)–5(h) illustrate graphically the behavior of translational velocity (*V*) versus boundary layer coordinate *z* for various involved parameters governing the flow field. For various values of Prandtl number (Pr), suction parameter (*S*), and radiation parameter (*F*), the profiles of translational velocity across boundary layer are shown in Figure 5(a). It is clearly evident that translational velocity decreases on increasing Pr because since Prandtl number is the ratio of kinematic viscosity to thermal diffusivity, so as Pr increases, the kinematic viscosity of the fluid dominates the thermal diffusivity of the fluid which leads to decreasing of the velocity of the flow field. Moreover, it is noticed that velocity first increases in the region adjacent to the plate and then decreases on moving away from the plate with increase in the suction parameter (*S*) showing the suction has a stabilizing effect on the flow field. This figure also incorporates the fact that radiation (*F*) tends to accelerate the translational velocity throughout the boundary layer region. Physically, it is true, as higher radiation occurs when temperature is higher and ultimately the velocity rises. The velocity distribution attains maximum value in the neighborhood of the wall and then decrease to approach the free stream value. The effect of heat absorption parameter on translational velocity (*V*) is depicted in Figure 5(b) and it is found that velocity reduces due to the presence of heat absorption parameter (*Q*).

Figure 5(c) incorporates the influence of magnetic parameter (*M*) and hall parameter (*m*) on the translational velocity profiles (*V*). As expected, the application of the transverse magnetic field retards the fluid motion. This phenomenon has an excellent agreement with the physical fact that the presence of transverse magnetic field in an electrically conducting fluid always generates a resistive type of force called Lorentz force which is similar to drag force and hence serves to decelerate the flow. As such the magnetic field is an effective regulatory mechanism for the flow regime. Form this figure it is also found that Hall currents (*m*) tends to accelerate the fluid velocity throughout the boundary layer region which is consistent with the fact that Hall currents induces flow in the flow field.

The combined effect of thermal and concentration buoyancy forces on the translational velocity are depicted in Figure 5(d). It is evident from this figure that with an increase in Grashof number (Gr) and modified Grashof number (Gm), which is a measure of thermal and concentration buoyancy forces, there is a substantial growth in the momentum boundary layer for the same reasons as explained earlier in this section.  Figure 5(e) depicts the effect of rotational parameter (*R*) on the fluid velocity and it is perceived that rotation tends to retard fluid velocity throughout the flow field. This is due to the reason that Coriolis force is dominant in the region near to the axis of rotation.

Variation of translational velocity profiles for different values of Soret parameter (Sr), Schmidt number (Sc), and chemical reaction (*α*) are displayed in Figure 5(f). The comparison of the curves shows that the velocity of the flow field decreases due to an increase in Schimdt number and Soret number. It is also observed from this figure that velocity decreases during the destructive reaction (*α* < 0).

Figure 5(g) depicts the influence of viscosity ratio (Δ) and permeability parameter (*K*) on the translational velocity (*V*). For different values of permeability parameter this figure shows that velocity increases with increasing values of *K* while an increasing viscosity ratio (Δ) results in an enhancement of the total viscosity in fluid flow because Δ is directly proportional to vortex viscosity which makes the fluid more viscous and so weakens the convection currents and hence the velocity decreases. This phenomenon has a good agreement with the physical realities.

Figure 5(h) incorporates the effect of slip or rarefaction parameter (*h*) and material parameter (*λ*) on the translational velocity (*V*). It is observed that an increase in the values of rarefaction parameter result in an enhancement of the flow field inside the boundary layer. This behavior is readily understood from the velocity slip condition at the surface (23). The case when *h* = 0 corresponds to the no slip condition and in the present case it reduces to the case when the plate moves with constant velocity in the longitudinal direction. The effects are more visible in the region near to the plate and afterwards it fall slowly and steadily to its free stream value as *z* → *∞*. Lastly the velocity decreases with increasing material parameter (*λ*). In Figures 5(a)–5(h) we observe that the velocity become maximum in the vicinity of the plate and then decreases away from the plate and finally takes asymptotic values far away from the plate.

**Figure 6:**
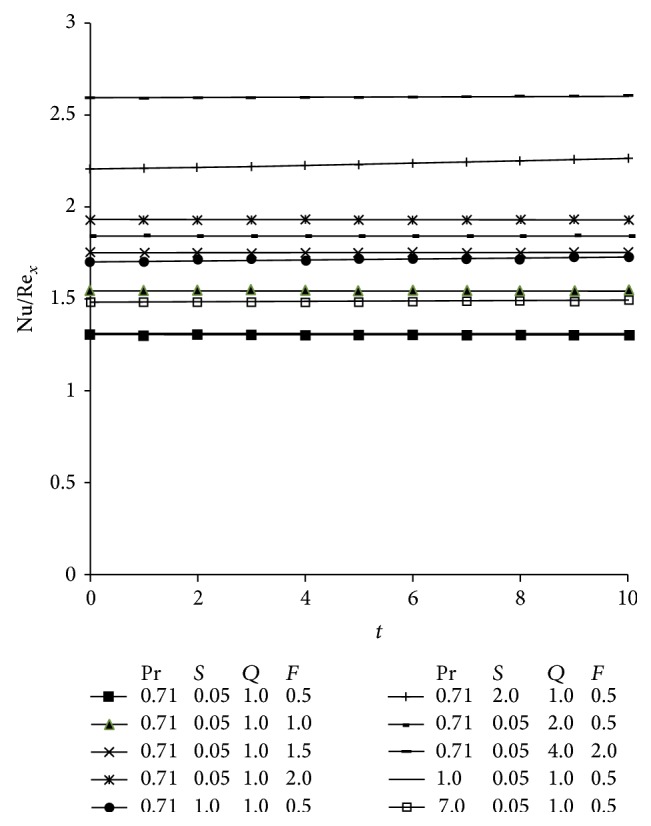
Nusselt number for different values of Prandtl number (Pr), suction parameter (*S*), heat absorption parameter (*Q*), and radiation parameter (*F*) taking *A* = 3, *t* = 1, and *δ* = 0.1.

The numerical values of Nusselt number computed from the analytical solution given in (39) are presented graphically versus time (*t*) in Figure 6 for various values of Prandtl number (Pr), suction parameter (*S*), heat absorption parameter (*Q*), and radiation parameter (*F*). It is noteworthy that the Prandtl number, suction parameter, heat absorption parameter, and radiation parameter enhance the rate of heat transfer at the surface of the plate. The reason behind this phenomenon is explained earlier in the text. The rate of heat transfer is more for water (Pr = 7.0) than that of air (Pr = 0.71).

**Figure 7:**
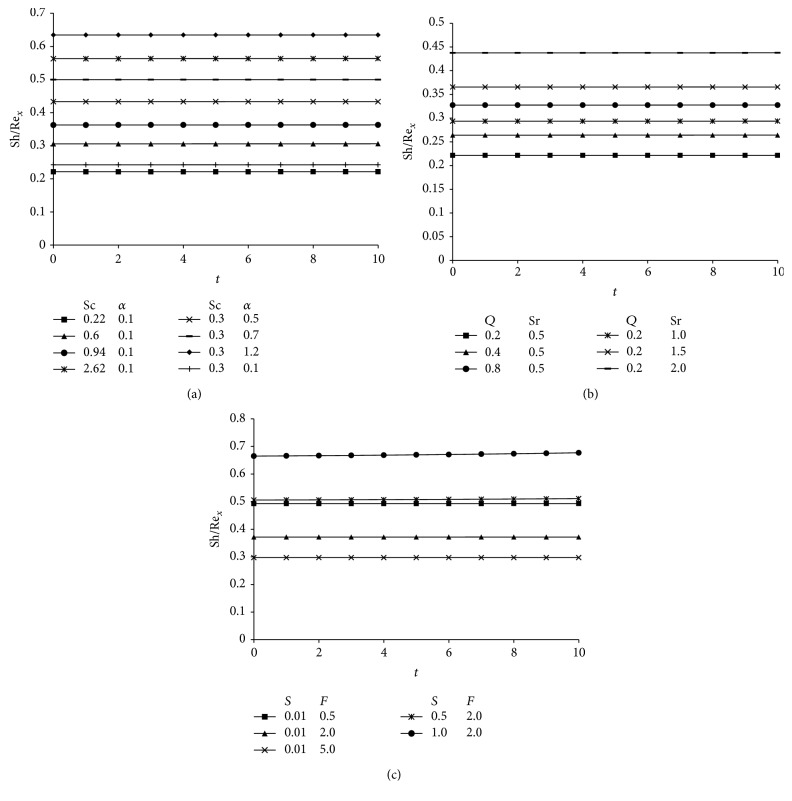
(a) Sherwood number showing the variation for Schmidt number (Sc) and chemical reaction parameter (*α*) taking Pr = 0.71, *S* = 0.01, *F* = 2, *Q* = 1, *δ* = 0.1, *t* = 1, and *A* = 3. (b) Sherwood number showing the variation for heat absorption parameter (*Q*) and Soret number (Sr) taking Pr = 0.71, *S* = 0.01, *F* = 2, Sc = 0.22, *δ* = 0.1, *t* = 1, and *A* = 3. (c) Sherwood number showing the variation for suction parameter (*S*) and radiation parameter (*F*) taking Pr = 0.71, Sr = 0.5, Sc = 0.3, *Q* = 1, *δ* = 0.1, *t* = 1, and *A* = 3.

Figures 7(a)–7(c) display the concentration gradient—*C*′(0) at the porous plate versus time (*t*). From all these figures it is analyzed that Sherwood number increase with an increase in Schmidt number (Sc), chemical reaction parameter (*α*), Soret number (Sr), suction parameter (*S*), heat absorption parameter (*Q*), and radiation parameter (*F*). As time progresses the Sherwood number remains unaltered.

**Figure 8:**
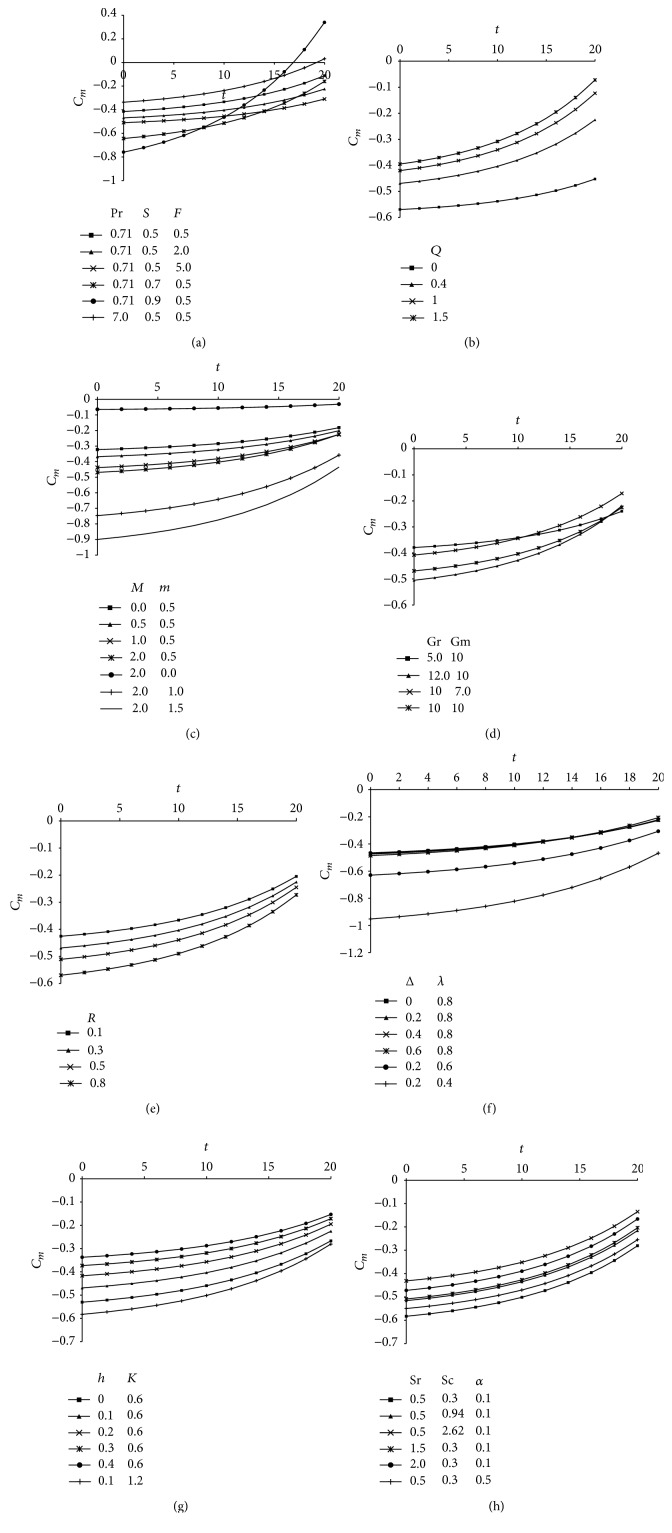
(a) Couple stress showing the variation of Prandtl number (Pr), suction parameter (*S*), and radiation parameter (*F*) taking *Q* = 0.4, *α* = 0.1, *δ* = 0.1, *t* = 1, *A* = 3, Sr = 0.5, Sc = 0.3, *M* = 2, *m* = 0.5, Gr = 10, Gm = 10, *R* = 0.3, *h* = 0.1, *K* = 0.6, Δ = 0.2, and *λ* = 0.8. (b) Couple stress showing the variation of heat absorption parameter (*Q*) taking Pr = 0.71, *S* = 0.5, *F* = 2, *α* = 0.1, *δ* = 0.1, *t* = 1, *A* = 3, Sr = 0.5, Sc = 0.3, *M* = 2, *m* = 0.5, Gr = 10, Gm = 10, *R* = 0.3, *h* = 0.1, *K* = 0.6, Δ = 0.2, and *λ* = 0.8. (c) Couple stress showing the variation of magnetic parameter (*M*) and Hall parameter (*m*) taking Pr = 0.71, *S* = 0.5, *F* = 2, *Q* = 0.4, *α* = 0.1, *δ* = 0.1, *t* = 1, *A* = 3, Sr = 0.5, Sc = 0.3, Gr = 10, Gm = 10, *R* = 0.3, *h* = 0.1, *K* = 0.6, Δ = 0.2, and *λ* = 0.8. (d) Couple stress showing the variation of Grashof number (Gr) and modified Grashof number (Gm) taking Pr = 0.71, *S* = 0.5, *F* = 2, *Q* = 0.4, *α* = 0.1, *δ* = 0.1, *t* = 1, *A* = 3, Sr = 0.5, Sc = 0.3, *M* = 2, *m* = 0.5, *R* = 0.3, *h* = 0.1, *K* = 0.6, Δ = 0.2, and *λ* = 0.8. (e) Couple stress showing the variation of rotational parameter (*R*) taking Pr = 0.71, *S* = 0.5, *F* = 2, *Q* = 0.4, *α* = 0.1, *δ* = 0.1, *t* = 1, *A* = 3, Sr = 0.5, Sc = 0.3, *M* = 2, *m* = 0.5, Gr = 10, Gm = 10, *h* = 0.1, *K* = 0.6, Δ = 0.2, and *λ* = 0.8. (f) Couple stress showing the variation of viscosity ratio (Δ) and material parameter (*λ*) taking Pr = 0.71, *S* = 0.5, *F* = 2, *Q* = 0.4, *α* = 0.1, *δ* = 0.1, *t* = 1, *A* = 3, Sr = 0.5, Sc = 0.3, *M* = 2, *m* = 0.5, Gr = 10, Gm = 10, *R* = 0.3, *h* = 0.1, and *K* = 0.6. (g) Couple stress showing the variation of slip parameter (*h*) and permeability parameter (*K*) taking Pr = 0.71, *S* = 0.5, *F* = 2, *Q* = 0.4, *α* = 0.1, *δ* = 0.1, *t* = 1, *A* = 3, Sr = 0.5, Sc = 0.3, *M* = 2, *m* = 0.5, Gr = 10, Gm = 10, *R* = 0.3, Δ = 0.2, and *λ* = 0.8. (h) Couple stress showing the variation of Soret parameter (Sr), Schmidt number (Sc), and chemical reaction parameter (*α*) taking Pr = 0.71, *S* = 0.5, *F* = 2, *Q* = 0.4, *δ* = 0.1, *t* = 1, *A* = 3, *M* = 2, *m* = 0.5, Gr = 10, Gm = 10, *R* = 0.3, *h* = 0.1, *K* = 0.6, Δ = 0.2, and *λ* = 0.8.

**Figure 9:**
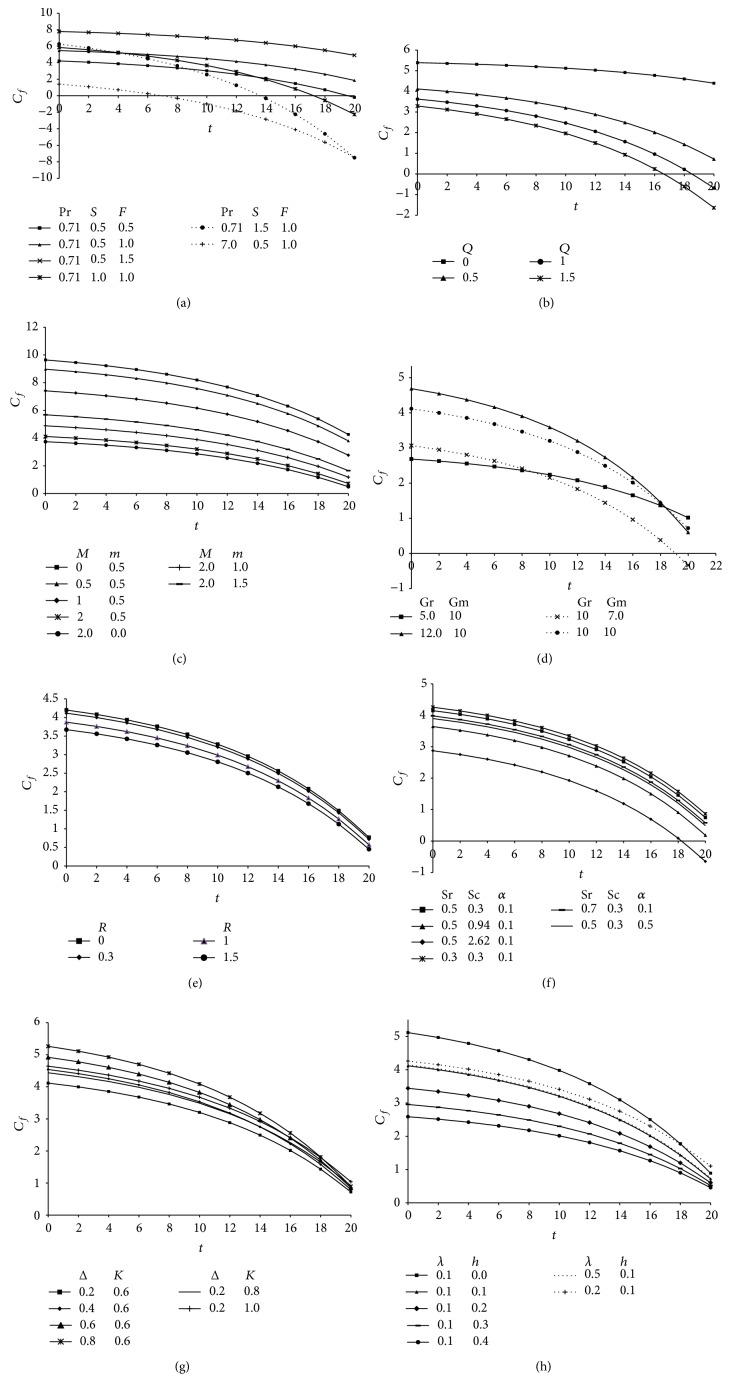
(a) Skin friction profiles showing the variation of Prandtl number (Pr), suction parameter (*S*), and radiation parameter (*F*) taking *Q* = 0.5, *α* = 0.1, *δ* = 0.1, *t* = 1, *A* = 3, Sr = 0.5, Sc = 0.3, *M* = 2, *m* = 0.5, Gr = 10, Gm = 10, *R* = 0.3, *h* = 0.1, *K* = 0.6, Δ = 0.2, and *λ* = 0.8. (b) Skin friction profiles showing the variation of heat absorption parameter (*Q*) taking Pr = 0.71, *S* = 0.5, *F* = 1, *α* = 0.1, *δ* = 0.1, *t* = 1, *A* = 3, Sr = 0.5, Sc = 0.3, *M* = 2, *m* = 0.5, Gr = 10, Gm = 10, *R* = 0.3, *h* = 0.1, *K* = 0.6, Δ = 0.2, and *λ* = 0.8. (c) Skin friction profiles showing the variation of magnetic parameter (*M*) and Hall parameter (*m*) taking Pr = 0.71, *S* = 0.5, *F* = 1, *Q* = 0.5, *α* = 0.1, *δ* = 0.1, *t* = 1, *A* = 3, Sr = 0.5, Sc = 0.3, Gr = 10, Gm = 10, *R* = 0.3, *h* = 0.1, *K* = 0.6, Δ = 0.2, and *λ* = 0.8. (d) Skin friction profiles showing the variation of Grashof number (Gr) and modified Grashof number (Gm) taking Pr = 0.71, *S* = 0.5, *F* = 1, *Q* = 0.5, *α* = 0.1, *δ* = 0.1, *t* = 1, *A* = 3, Sr = 0.5, Sc = 0.3, *M* = 2, *m* = 0.5, *R* = 0.3, *h* = 0.1, *K* = 0.6, Δ = 0.2, and *λ* = 0.8. (e) Skin friction profiles showing the variation of rotational parameter (*R*) taking Pr = 0.71, *S* = 0.5, *F* = 1, *Q* = 0.5, *α* = 0.1, *δ* = 0.1, *t* = 1, *A* = 3, Sr = 0.5, Sc = 0.3, *M* = 2, *m* = 0.5, Gr = 10, Gm = 10, *h* = 0.1, *K* = 0.6, Δ = 0.2, and *λ* = 0.8. (f) Skin friction profiles showing the variation of Soret parameter (Sr), Schimdt number (Sc), and chemical reaction parameter (*α*) taking Pr = 0.71, *S* = 0.5, *F* = 1, *Q* = 0.5, *δ* = 0.1, *t* = 1, *A* = 3, *M* = 2, *m* = 0.5, Gr = 10, Gm = 10, *R* = 0.3, *h* = 0.1, *K* = 0.6, Δ = 0.2, and *λ* = 0.8. (g) Skin friction profiles showing the variation of viscosity ratio (Δ) and permeability parameter (*K*) taking Pr = 0.71, *S* = 0.5, *F* = 2, *Q* = 0.5, *α* = 0.1, *δ* = 0.1, *t* = 1, *A* = 3, Sr = 0.5, Sc = 0.3, *M* = 2, *m* = 0.5, Gr = 10, Gm = 10, *R* = 0.3, *h* = 0.1, and *λ* = 0.1. (h) Skin friction profiles showing the variation of slip parameter (*h*) and material parameter (*λ*) taking Pr = 0.71, *S* = 0.5, *F* = 2, *Q* = 0.5, *α* = 0.1, *δ* = 0.1, *t* = 1, *A* = 3, Sr = 0.5, Sc = 0.3, *M* = 2, *m* = 0.5, Gr = 10, Gm = 10, *R* = 0.3, Δ = 0.2, and *K* = 0.6.

The variation of couple stress coefficient (*C*_*m*_) for various involved parameters is displayed in Figures 8(a)–8(h) versus time (*t*).  Figure 8(a) exhibits that couple stress coefficient decreases with increasing values of radiation parameter (*F*) and suction parameter (*S*) it increases with increasing values of Prandtl number (Pr). The effect of heat absorption parameter (*Q*) on *C*_*m*_ is shown in Figure 8(b) and it is found that couple stress coefficient enhances with the rise in the values of *Q*. From Figures 8(c)–8(f) it is apparent that the effect of increasing values of magnetic parameter (*M*), hall parameter (*m*), Grashof number (Gr), modified Grashof number (Gm), Rotational parameter (*R*) and viscosity ratio (Δ) are to decrease the values of couple stress coefficient whereas reverse effect is found on increasing the values of material parameter (*λ*).  Figure 8(g) shows a substantial growth in couple stress coefficient with increasing values of slip parameter (*h*) while reverse happen for increasing values of permeability parameter (*K*). Finally, the Schmidt number (Sc), Soret number (Sr), and chemical reaction parameter (*α*) have the tendency to increase couple stress coefficient and this is clearly visible in Figure 8(h). From all these figures from Figures 8(a) to 8(h) it is understandable that as time progresses couple stress coefficient (*C*_*m*_) is getting enhanced whereas from Figures 9(a) to 9(h) it is visible that skin friction coefficient (*C*_*f*_) is getting suppressed for increasing values of time (*t*).

The skin friction is an important phenomenon which characterizes the frictional drag at the solid surface, so the numerical values of skin friction coefficient (*C*_*f*_) computed from (33) is presented in Figures 9(a)–9(h) taking different values of *F*, *S*, Pr, *Q*, *M*, *m*, Gr, Gm, *R*, Sc, Sr, *α*, Δ, *λ*, *K*, and *h*. The skin friction coefficient increases with increasing values of radiation parameter while it decreases with increase in suction parameter, Prandtl number, and heat absorption parameter and this fact is depicted in Figures 9(a) and 9(b). It is noticed from Figure 9(c) that the skin friction coefficient is reduced due to an increase in magnetic field strength as expected, since the applied magnetic field tends to impede the flow motion and thus reduces the surface friction force while the hall parameter tends to increase the skin friction.  Figure 9(d) demonstrates the growth in skin friction for increasing values of thermal buoyancy parameter (Gr) and modified Grashof number (Gm) because an increase in buoyancy effect in mixed convection flow leads to an acceleration of the fluid flow which increases the friction factor. An opposite trend is observed for increasing values of rotational parameter; that is, *C*_*f*_ decreases with an increase in *R*. The influence of Schmidt number (Sc), Soret number (Sr), and chemical reaction parameter (*α*) on skin friction coefficient is exhibited in Figure 9(f) and all these parameters tend to retard the surface friction forces. Finally, Figures 9(g) and 9(h) exhibit a significant growth in *C*_*f*_ with increasing values of viscosity ratio, permeability parameter, and slip parameter while reverse happens with increasing material parameter.

## 5. Conclusion

The governing equations were solved analytically using perturbation technique. The effects of various parameters on the temperature *θ*, concentration *C*, translational velocity *V*, microrotation *ω*, skin-friction *C*_*f*_, Nusselt number, and Sherwood number are examined. From the present calculations, we arrive at the following findings.

- Thermal radiation tends to enhance fluid temperature whereas there is a decrement in fluid temperature with an increase of Prandtl number, suction parameter, and heat absorption parameter.
- The species concentration profiles decrease at all points in the flow field with an increase in Schmidt number, chemical reaction parameter, heat generation parameter, Soret number, and suction parameter but are enhanced with an increase in radiation parameter while these physical quantities show reverse trend for Sherwood number.
- Thermal radiation, magnetic parameter, hall parameter, and permeability parameter tend to enhance the microrotation distribution whereas these physical quantities have reverse effect on couple stress coefficient.
- Microrotation profiles decrease with an increase in Prandtl number, material parameter, slip parameter, Soret number, Schmidt number, and chemical reaction parameter whereas these physical quantities have reverse effect on couple stress coefficient.
- Microrotation profiles and couple stress coefficient decrease with an increase in suction parameter, rotation parameter, and viscosity ratio.
- Thermal radiation parameter, permeability parameter, and slip parameter tend to enhance the translational velocity profiles throughout the boundary layer region and the skin-friction coefficient.
- Prandtl number, magnetic parameter, suction parameter, rotation parameter, Soret number, Schmidt number, chemical reaction parameter, viscosity ratio, and material parameter tend to enhance both translational velocity profiles and skin-friction coefficient.
- Slip parameter increases the translational velocity profiles but decreases the skin-friction coefficient.
- Thermal radiation parameter, Prandtl number, suction parameter, and heat absorption parameter tend to enhance dimensionless rate of heat transfer, that is, Nusselt number.

## Appendix

The exponential indices in 28–31 are given by

(A.1) r1=3Pr⁡S+3Pr⁡S2+123+4FQ23+4F,r2=3Pr⁡S+3Pr⁡S2+123+4FQ+Pr⁡δ23+4F,r3=SSc+SSc2+4αSc2,r4=SSc+SSc2+4Scα+δ2,r5=S+S2+41+Δa121+Δ,r6=S+S2+4λδ2λ,r7=S+S2+41+Δa221+Δ

and the coefficients in 28–31 are given by

(A.2) a1=M21+m2+1K+iR+mM21+m2, a2=M21+m2+1K+δ+iR+mM21+m2, B1=3Pr⁡SAr13+4Fr12−3Pr⁡Sr1−3Q+Pr⁡δ, B2=Srr12r12−SScr1−αSc,  B3=1−B2, B4=SScAB3r3r32−SScr3−Scα+δ, B5=SScAB2r1−SrScB1r12r12−SScr1−Scα+δ, B6=SrScB1r22r22−SScr2−Scα+δ, B7=−B4−B5−B6, B8=−Gr−GmB21+Δr12−Sr1−a1, B9=−GmB31+Δr32−Sr3−a1,B10=ΔSλr5+B8r1−r5+B9r3−r521+ΔS2−S2λ−a1λ21+hr5−ΔSS−r5λ,B11=1−B8(1+hr1)−B9(1+hr3)−B101+hS/λ1+hr5,   B12 =−S2AD1λδ,   B13 =−GrB1−GmB5−AB81+Δr12−Sr1−a2,   B14 =−GrB1−GmB61+Δr22−Sr2−a2,   B15 =−GmB71+Δr42−Sr4−a2,   B16 =−GmB4−AB91+Δr32−Sr3−a2,   B17 =−AB111+Δr52−Sr5−a2,   B18 =−AB10+iΔS/λB121+ΔS/λ2−S2/λ−a2,B19=Δr6B13r1−r7+B14r2−r7+B15r4−r7Sλ−r7+B18S/λ−r7+−i21+hr7B12          +B16r3−r7+B17r5−r7          + B18Sλ−r7+−i21+hr7B12Δr6B13r1−r7+B14r2−r7+B15r4−r7Sλ−r7+B18S/λ−r7+−i21+hr7B12    ×21+Δr12−Sr1−a21+hr7−Δr6r6−r7−1      21+Δr12−Sr1−a21+hr7−Δr6r6−r7−1 −Δr6r6−r7−1,B20=−1+hr1B13+1+hr2B14+1+hr4B15+1+hSλB18+1+hr6B19       +1+hr3B16+1+hr5B17       +1+hSλB18+1+hr6B19   ×1+hr7−1,  D1 =i2−B11r5−B8r1−B9r3−B10Sλ,D2=−i2   ×B20r7+B13r1+B14r2+B15r4+B16r3+B17r5+B18Sλ+B19r6     + B17r5+B18Sλ+B19r6−B12.

## Nomenclature

*a*_*R*_: Rosseland mean absorption coefficient
*A*: Positive constant
*B*_0_: Applied magnetic field
*C*: Concentration of the solute in dimensionless form
*C*^*^: Concentration of the solute in the fluid
*C*_*w*_^*^: Concentration of the solute near the plate
*C*_*∞*_^*^: Concentration of the solute far away from the plate
*C*_*p*_: Specific heat at constant pressure
*C*_*f*_: Skin-friction coefficient
*C*_*m*_: Couple stress coefficient
*D*_*m*_: Chemical molecular diffusivity
en_*e*_: Electron charger
*F*: Radiation parameter
*g*: Acceleration due to gravity
Gr: Grashof number
Gm: Modified Grashof number
*h*: Rarefaction parameter
$\vec{H}$: Magnetic field strength
*H*_0_: Externally applied transverse magnetic field
*i*: Imaginary unit
*j*: Microinertia density
$\vec{J}$: Current strength
*K*^*^: Permeability of the porous medium
*K*: Nondimensional permeability of the porous medium
*k*: Thermal conductivity of the fluid
*K*_*T*_: Thermal diffusion ratio
*M*: Magnetic parameter
*m*: Hall parameter
*n*_*e*_: Number density of the electron
*P*_*e*_: Electron pressure
*Pr*⁡: Prandtl number
*Q*: Heat absorption parameter
*q*_*r*_: Radiative heat flux
*R*: Rotational parameter
*R*_*C*_: Chemical reaction rate constant
*S*: Suction parameter
Sc: Schmidt number
Sr: Soret number
*t*: Dimensionless time
*t*^*^: Time
*T*_*m*_: Mean fluid temperature
*T*: Temperature of the fluid near the plate
*T*_*w*_: Temperature of the plate
*T*_*∞*_: Temperature of the fluid far away from the plate
*U*_*r*_: Plate velocity in the direction of flow
$\vec{V}$: Velocity vector
(*u*^*^, *v*^*^, *w*^*^): Components of dimensional velocities along *x* ^*^-, *y* ^*^-, and *z* ^*^- direction, respectively
(*u*, *v*, *w*): Components of Dimensionless velocities along *x*-, *y*-, and *z*- direction, respectively
(*x*^*^, *y*^*^, *z*^*^): Cartesian coordinates
(*x*, *y*, *z*): Dimensionless Cartesian coordinates.

### Greek Symbols

*δ:*: Exponential index
*δ*^*^: Dimensionless exponential index
*α*: Chemical reaction parameter
*β*_*T*_: Coefficient of volumetric thermal expansion of the fluid
*β*_*C*_: Volumetric coefficient of expansion with concentration
Δ: Dimensionless viscosity ratio
*ν*: Kinematic viscosity
*ν*_*r*_: Kinematic rotational viscosity
Λ: Spin-gradient viscosity
*Ω*: Angular velocity
*λ*: Dimensionless material parameter
*μ*: Coefficient of viscosity
*μ*_*e*_: Magnetic permeability
*σ*: Electrical conductivity of the fluid
*σ*^*^: Stefan-Boltzmann constant
*ρ*: Density of the fluid
*τ*_*e*_: Electron collision time
*ω*_*e*_: Electron frequency
(*ω*_1_, *ω*_2_): Microrotation components
*ω*: Dimensionless microrotation profile
*θ*: Dimensionless temperature of the plate.
